# Caudate functional networks influence brain structural changes with aging

**DOI:** 10.1093/braincomms/fcae116

**Published:** 2024-04-09

**Authors:** Silvia Basaia, Matteo Zavarella, Giulia Rugarli, Giacomo Sferruzza, Camilla Cividini, Elisa Canu, Laura Cacciaguerra, Marco Bacigaluppi, Gianvito Martino, Massimo Filippi, Federica Agosta

**Affiliations:** Neuroimaging Research Unit, Division of Neuroscience, IRCCS San Raffaele Scientific Institute, 20132 Milan, Italy; Neuroimaging Research Unit, Division of Neuroscience, IRCCS San Raffaele Scientific Institute, 20132 Milan, Italy; Vita-Salute San Raffaele University, 20132 Milan, Italy; Neuroimaging Research Unit, Division of Neuroscience, IRCCS San Raffaele Scientific Institute, 20132 Milan, Italy; Vita-Salute San Raffaele University, 20132 Milan, Italy; Vita-Salute San Raffaele University, 20132 Milan, Italy; Neurology Unit, IRCCS San Raffaele Scientific Institute, 20132 Milan, Italy; Neuroimmunology Unit, Division of Neuroscience, IRCCS San Raffaele Scientific Institute, 20132 Milan, Italy; Neuroimaging Research Unit, Division of Neuroscience, IRCCS San Raffaele Scientific Institute, 20132 Milan, Italy; Neuroimaging Research Unit, Division of Neuroscience, IRCCS San Raffaele Scientific Institute, 20132 Milan, Italy; Neuroimaging Research Unit, Division of Neuroscience, IRCCS San Raffaele Scientific Institute, 20132 Milan, Italy; Neurology Unit, IRCCS San Raffaele Scientific Institute, 20132 Milan, Italy; Neuroimmunology Unit, Division of Neuroscience, IRCCS San Raffaele Scientific Institute, 20132 Milan, Italy; Vita-Salute San Raffaele University, 20132 Milan, Italy; Neuroimmunology Unit, Division of Neuroscience, IRCCS San Raffaele Scientific Institute, 20132 Milan, Italy; Neuroimaging Research Unit, Division of Neuroscience, IRCCS San Raffaele Scientific Institute, 20132 Milan, Italy; Vita-Salute San Raffaele University, 20132 Milan, Italy; Neurology Unit, IRCCS San Raffaele Scientific Institute, 20132 Milan, Italy; Neurophysiology Service, IRCCS San Raffaele Scientific Institute, 20132 Milan, Italy; Neurorehabilitation Unit, IRCCS San Raffaele Scientific Institute, 20132 Milan, Italy; Neuroimaging Research Unit, Division of Neuroscience, IRCCS San Raffaele Scientific Institute, 20132 Milan, Italy; Vita-Salute San Raffaele University, 20132 Milan, Italy; Neurology Unit, IRCCS San Raffaele Scientific Institute, 20132 Milan, Italy

**Keywords:** connectomics, aging, caudate, structural MRI, subventricular zone

## Abstract

Neurogenesis decline with aging may be associated with brain atrophy. Subventricular zone neuron precursor cells possibly modulate striatal neuronal activity via the release of soluble molecules. Neurogenesis decay in the subventricular zone may result in structural alterations of brain regions connected to the caudate, particularly to its medial component. The aim of this study was to investigate how the functional organization of caudate networks relates to structural brain changes with aging. One hundred and fifty-two normal subjects were recruited: 52 young healthy adults (≤35 years old), 42 middle-aged (36 ≤ 60 years old) and 58 elderly subjects (≥60 years old). In young adults, stepwise functional connectivity was used to characterize regions that connect to the medial and lateral caudate at different levels of link-step distances. A statistical comparison between the connectivity of medial and lateral caudate in young subjects was useful to define medial and lateral caudate connected regions. Atrophy of medial and lateral caudate connected regions was estimated in young, middle-aged and elderly subjects using T_1_-weighted images. Results showed that middle-aged and elderly adults exhibited decreased stepwise functional connectivity at one-link step from the caudate, particularly in the frontal, parietal, temporal and occipital brain regions, compared to young subjects. Elderly individuals showed increased stepwise functional connectivity in frontal, parietal, temporal and occipital lobes compared to both young and middle-aged adults. Additionally, elderly adults displayed decreased stepwise functional connectivity compared to middle-aged subjects in specific parietal and subcortical areas. Moreover, in young adults, the medial caudate showed higher direct connectivity to the basal ganglia (left thalamus), superior, middle and inferior frontal and inferior parietal gyri (medial caudate connected region) relative to the lateral caudate. Considering the opposite contrast, lateral caudate showed stronger connectivity to the basal ganglia (right pallidum), orbitofrontal, rostral anterior cingulate and insula cortices (lateral caudate connected region) compared to medial caudate. In elderly subjects, the medial caudate connected region showed greater atrophy relative to the lateral caudate connected region. Brain regions linked to the medial caudate appear to be more vulnerable to aging than lateral caudate connected areas. The adjacency to the subventricular zone may, at least partially, explain these findings. Stepwise functional connectivity analysis can be useful to evaluate the role of the subventricular zone in network disruptions in age-related neurodegenerative disorders.

## Teaser

Basaia *et al*. report that brain structural changes are differently shaped by the medio-lateral gradient of connections with the caudate nucleus due to age-related periventricular changes, including vascular, degenerative and inflammatory alterations and a possible subventricular zone neurogenesis decay.

## Graphical Abstract

**Graphical Abstract:**
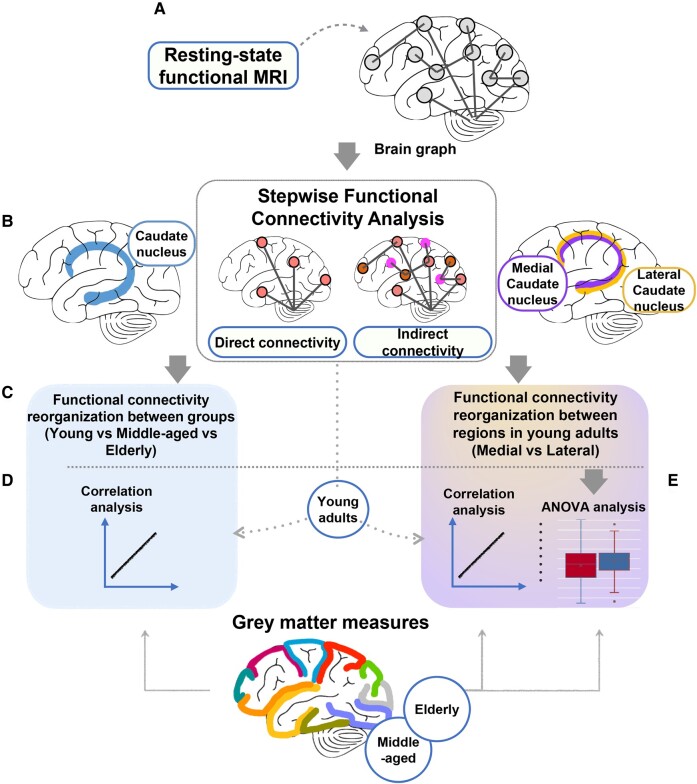

## Introduction

Alterations in caudate networks have long been recognized as a potentially important predictor of declining cognitive functioning in the aging human brain and neurodegenerative disorders.^1,2^ Reductions of dopamine receptors and transporters in aging are a reliable observation.^3^ Functional MRI (fMRI) studies demonstrated that caudate functional connectivity can predict change in memory over years in elderly individuals.^2^ Specifically, alterations in fronto-striatal connectivity, particularly with the caudate, play a significant role in episodic memory decline during aging, influencing different aspects of memory function compared to the hippocampus.^4,5^

Between 20 and 30 years old, generally, the human brain undergoes a period of maturation and consolidation of its functions that might bring to structural and functional connectivity changes. During this period, there is no significant alteration associated with aging.^6,7^ On the contrary, consistent literature has provided insights into the structural and functional changes that occur in the brains of individuals aged 60 and above. At a macroscopic level, the hippocampus and frontal lobes were among the most affected regions.^8^ The caudate nucleus is connected to the cortex through complex multi-synaptic circuits. Cortico-striatal projections are suggested to be organized according to both a proximity criterion (i.e. each cortical area innervates the closest striatal area)^9^ and a functional criterion (e.g. associative, sensorimotor and limbic).^10^ In previous fMRI studies, caudate connections have been explored using multiple subdivisions: anterior–posterior, medial–lateral or dorsal–ventral organization.^11^ A few recent studies suggested a relation between medial–lateral gradient and functional cortical association networks. Specifically, medial caudate was linked to the default mode network (DMN), while the lateral caudate was connected to the frontoparietal control brain networks.^2^ With normal aging, the relation between caudate subfields (including the medial–lateral gradient) and cortical association networks is less differentiated,^12^ in keeping with the appearance of cognitive decline.^2,12^

Degeneration of periventricular brain areas is common with normal aging, likely due to impaired transependymal bulk flow mechanisms^13^ but also age-related inflammatory changes, changes in vasculature and in circulating factors in the blood.^14,15^ In the adult brain, neural stem/precursors cells are present in areas known as neurogenic niches, like the subgranular zone located in the dentate gyrus of the hippocampus^16^ and the subventricular zone (SVZ) adjacent to the lateral ventricles^17^ and very close to the medial wall of the caudate nucleus. Neurogenic niches undergo age-related modifications, including changes in endothelium and astrocytes, and, as a result, SVZ may have a reduced number of neural precursor cells.^18,19^ Therefore, it is hypothesized that SVZ neural stem/precursor cells influence global brain aging through its relationship with caudate nucleus. However, the relation between neurodegeneration, caudate nucleus network and SVZ is yet to be completely understood. Recent evidence suggested that the SVZ neural stem/precursor cells exert a non-neurogenic physiological role on structural, electrophysiological and behavioural aspects of striatal function.^20^ Indeed, damage of the SVZ significantly correlated with caudate volume and cognitive impairment in patients with multiple sclerosis.^20^

Brain atrophic changes occur with aging.^21^ A co-localization of functional connectivity alterations and atrophy in clinically normal elderly individuals has been observed.^22^ Brain connectome topology and geometry have been suggested to shape the accumulation of biological damage and distribution of atrophy with aging.^23^ A novel graph theory metric based on stepwise functional connectivity (SFC) analysis^24^ allows for the mapping of connectivity patterns of brain ‘seed’ regions and considers direct and indirect connectivity routes. In this way, it might be possible to reveal how the functional organization of caudate networks relates to structural brain changes with normal aging and whether this relationship is potentially influenced by the proximity of the caudate nucleus to the periventricular brain regions. We hypothesized that the functional disruption of the caudate nucleus would predict brain atrophy of regions within the cortico-striatal network over the life span. We also expect that brain structural changes should be differently shaped by the medio-lateral gradient of connections with the caudate nucleus due to the age-related periventricular/SVZ degeneration. We applied SFC analysis, which maps the connectivity patterns of brain seed regions at different step (or ‘link-step’) distances from a seed region of interest,^24^ thus discriminating between alterations of one-step (direct) and longer-distance (indirect) connections. This framework for neuroimaging analyses has recently demonstrated great potential for the study of neurodegenerative disorders *in vivo*.^25^

## Materials and methods

For this prospective study, participants were recruited and evaluated at the IRCCS San Raffaele Scientific Institute (Milan, Italy), from 2017 to date, in the framework of an observational study. MRI scans were collected from all participants using a Philips Medical Systems Ingenia cx 3T scanner. MRI data were pre-processed and analysed at the Neuroimaging Research Unit, Division of Neuroscience, IRCCS San Raffaele Scientific Institute, Milan, Italy.

### Participants

One hundred and fifty-two Caucasian, right-handed healthy subjects were recruited by word of mouth. None of the participants had history of psychiatric or neurological disorder, drug or alcohol abuse or any systemic disease that might compromise cognitive function or blood flow (e.g. diabetes, untreated hypertension or cardiovascular disease). All participants scored in the normal range on the Mini-Mental Status Exam (MMSE, ≥27).^26^ Before participation, written informed consent was obtained from all subjects.

Participants were aged between 20 and 85 years. They were divided into three groups according to age: 52 young healthy adults (≤35 years old), 42 middle-aged (36 ≤ 60 years old) and 58 elderly (≥60 years old) subjects. At the study entry, all subjects performed a comprehensive neuropsychological and behavioural evaluation (see Supplementary material and Supplementary Table 1 for more details). In addition, each participant underwent a brain MRI scan, including 3D T_2_-weighted, 3D fluid-attenuated inversion recovery, 3D high-resolution T_1_-weighted sequences and T_2_*-weighted (GE-EPI) as resting-state fMRI (RS-fMRI) sequence (see Supplementary Table 5 for more details).

### Statistical analysis: demographic and cognitive data

Demographic and cognitive data were compared between young, middle-aged and elderly healthy adults using one-way ANOVA models (for continuous variables) or chi-square test (for categorical variables; see Table 1 and Supplementary Table 1 for more details). Cognitive data analysis was corrected for age, sex and education. A two-sided *P* < 0.05 was considered for statistical significance. *P*-values were adjusted for Bonferroni multiple comparisons. Analyses were performed using R Statistical Software (version 4.0.3; R Foundation for Statistical Computing, Vienna, Austria).

**Table 1:** Demographic variables in young, middle-aged and elderly subjects

	Young healthy subjects	Middle-aged subjects	Elderly subjects	*P*-value, young versus middle-aged subjects	*P*-value, young versus elderly subjects	*P*-value, middle-aged versus elderly subjects
*N*	52	42	58			
Age (years)	25.48 ± 3.08 (20.48–31.69)	53.78 ± 5.78 (36.82–60.46)	69.62 ± 5.11 (61.56–84.59)	<0.001	<0.001	<0.001
Sex (M/W)	28/24	15/27	19/39	0.06	0.03	0.46
Education (years)	15.60 ± 3.01 (8.00–24.00)	13.60 ± 3.30 (8.00–20.00)	11.38 ± 3.76 (5.00–18.00)	0.02	<0.001	0.01

Values are reported as mean ± standard deviation (range). Categorical variables are reported as frequency. Demographic data were compared between young healthy adults and middle-aged and elderly adults using one-way ANOVA models (for continuous variables) or chi-square test (for categorical variables). A two-sided *P* < 0.05 was considered for statistical significance. *P*-values were adjusted for Bonferroni multiple comparisons. M, men; *N*, number; W, women.

### MRI analysis

Prior to the pre-processing of MR images, T_2_-weighted sequence was used to identify/exclude the presence of vascular abnormalities, including white matter hyperintensities and lacunes.

#### SFC analysis

The pipeline applied for this study, including RS-fMRI pre-processing, functional connectome reconstruction and SFC implementation, has been recently described,^24,27^ and further details are reported in the Supplementary material. Based on such framework, SFC patterns are topographically dissimilar between consecutive maps from steps one to three and become stable for link-step distances above four.^24,27^ For such reason, in our results, we show maps up to four steps. In all healthy subject groups, we will refer to functional connectivity at one-link step as direct connectivity and to functional connectivity at subsequent steps (two to four) as indirect connectivity. SFC analysis requires the identification of a seed region of interest. Since this study aimed to investigate how the functional organization of caudate network relates to structural brain changes with aging, right and left caudate were considered as a bilateral seed. Bilateral caudate regions of interest were obtained from 3D T_1_-weighted images (see below). To identify ‘normally’ connected regions with the caudate, four SFC maps for each of the four steps were obtained averaging all the young healthy subject maps. Then, aging-related caudate SFC changes were explored comparing SFC maps across different link-step distances from one to four (i.e. SFC maps one to four) between young versus middle-aged and elderly subjects. Voxel-wise analyses were performed using general linear models as implemented in SPM12. Whole-brain two-sample *t*-test comparisons between groups were performed, including sex and education as covariates. A threshold-free cluster enhancement method, combined with nonparametric permutation testing (5000 permutations) as implemented in the Computational Anatomy Toolbox 12 (CAT12, http://www.neuro.uni-jena.de/cat/), was used to detect statistically significant differences at *P* < 0.05, family-wise error corrected.

In order to define brain regions ‘normally’ functionally connected with the caudate subcomponents, in healthy young subjects, the caudate was divided into two distinct parts due to the assumed different impact of aging on the two sections, considering the distance from the SVZ, i.e. medial and lateral caudate nuclei encompassing, respectively, the medial and lateral part of the caudate. Since the SVZ lies along the ventricular border, the medial caudate seed included all the voxels that are adjacent to the ventricles. Automatic segmentation was performed using MATLAB software. Medial and lateral caudate SFC maps (i.e. SFC maps one to four) were defined and compared using voxel-wise analysis with whole-brain paired *t*-test (see above).^20,28^

For visual purposes, SFC maps resulting from statistical analysis were projected onto the cerebral hemispheres of the Population-Average, Landmark- and Surface-based (PALS) surface (PALS-B12) provided with Caret software^29^ using the ‘enclosing voxel algorithm’ and ‘multifiducial mapping’ settings.

#### Cortical thickness measurement and grey matter volumes

Cortical reconstruction and estimation of cortical thickness were performed on the 3D high-resolution T_1_-weighted using the FreeSurfer image analysis suite, version 5.3 (http://surfer.nmr.mgh.harvard.edu/). The process involved registration to Talairach space, normalization of intensity and an automatic skull stripping to remove extra-cerebral structures. After such processing, images were segmented into grey matter (GM), white matter (WM) and CSF. The cerebral hemispheres were separated. Subcortical regions were not included in such analysis due to the low accuracy of such framework for deep cerebral structures. The WM/GM boundary was automatically delineated, and the surface was deformed following the intensity gradients to optimally recognize WM/GM and GM/CSF borders. Thus, WM and pial surface was obtained. The segmentation results were visually inspected and, if necessary, edited manually. Finally, the cerebral cortex was parcellated into 68 cortical regions based on Desikan atlas, and mean cortical thickness was calculated per each region as the average shortest distance between WM borders and pial surface.

FMRIB’s Integrated Registration and Segmentation Tool (FIRST) in FSL (http://www.fmrib.ox.ac.uk/fsl/first/index.html) was applied to 3D high-resolution T_1_-weighted images of each subject and used to automatically segment GM regions, i.e. caudate, pallidum, putamen, thalamus, amygdala and hippocampus, bilaterally. Mean GM volumes were calculated and multiplied by the normalization factor derived from SIENAx to correct for subject head size (http://www.Fmrib.ox.ac.uk/fsl/sienax/index.html).

Each cortical thickness value and GM volume of middle-aged and elderly subjects were then standardized. Mean and standard deviation values of each region in young subjects were calculated. For each middle-aged and elderly adult, the raw value of each region was considered, and, then, the mean value in the young subjects of the same region was subtracted to the raw value. The result was divided by the standard deviation obtained from the same region in the young subjects, according to the following equation:


$$Z\;=\;\frac{x_{\mathrm{OC}}-\;\mu_{\mathrm{YC}}}{\sigma_{\mathrm{YC}}},$$

where *Z* is the standardized value of each region in old subjects; χ_OC_ is the raw value of a region of old subjects, *μ*_YC_ is the mean cortical thickness in young subjects of the considered region and σ_YC_ is the standard deviation of the considered region in young subjects.

ANOVA analysis was performed to evaluate differences of standardized cortical thickness values and GM volumes among regions more connected to the medial caudate than the lateral and regions more connected to the lateral than the medial (see between-region differences in the previous ‘SFC analysis’ section for details) in middle-aged and elderly healthy subjects.

#### Spatial similarities between caudate SFC in young subjects and GM measures with aging

In healthy young subjects, a combined version of all SFC one to four maps into one single map (combined SFC map) was obtained for each seed (whole caudate and then medial and lateral caudate). The values of each voxel in the SFC combined map were set to the number of step (from 1 to 4, 1 = functionally closer to the caudate nuclei seed; 4 = functionally distant from caudate nuclei seed) in which the functional connectivity was maximized. A mean combined SFC map of young subjects was obtained by averaging all the young healthy subject maps.

To investigate spatial similarities between the combined SFC map in young subjects and regional cortical thickness values and GM volumes in middle-aged and elderly adults, SFC metrics of young healthy subjects were converted from the voxel-level spatial resolution to region levels (68 Desikan atlas and subcortical regions). Regional SFC metrics of young subjects were then correlated to regional standardized GM measures (cortical and subcortical regions) of middle-aged and elderly subjects, separately, using Pearson’s correlation (R Statistical Software). Furthermore, we tested the correlation between SFC of each region at the first-link step in young subjects, as a measure of the strength of direct connectivity, and the regional standardized GM measures (cortical and subcortical regions) of middle-aged and elderly subjects, separately.

## Results

**Figure 1:**
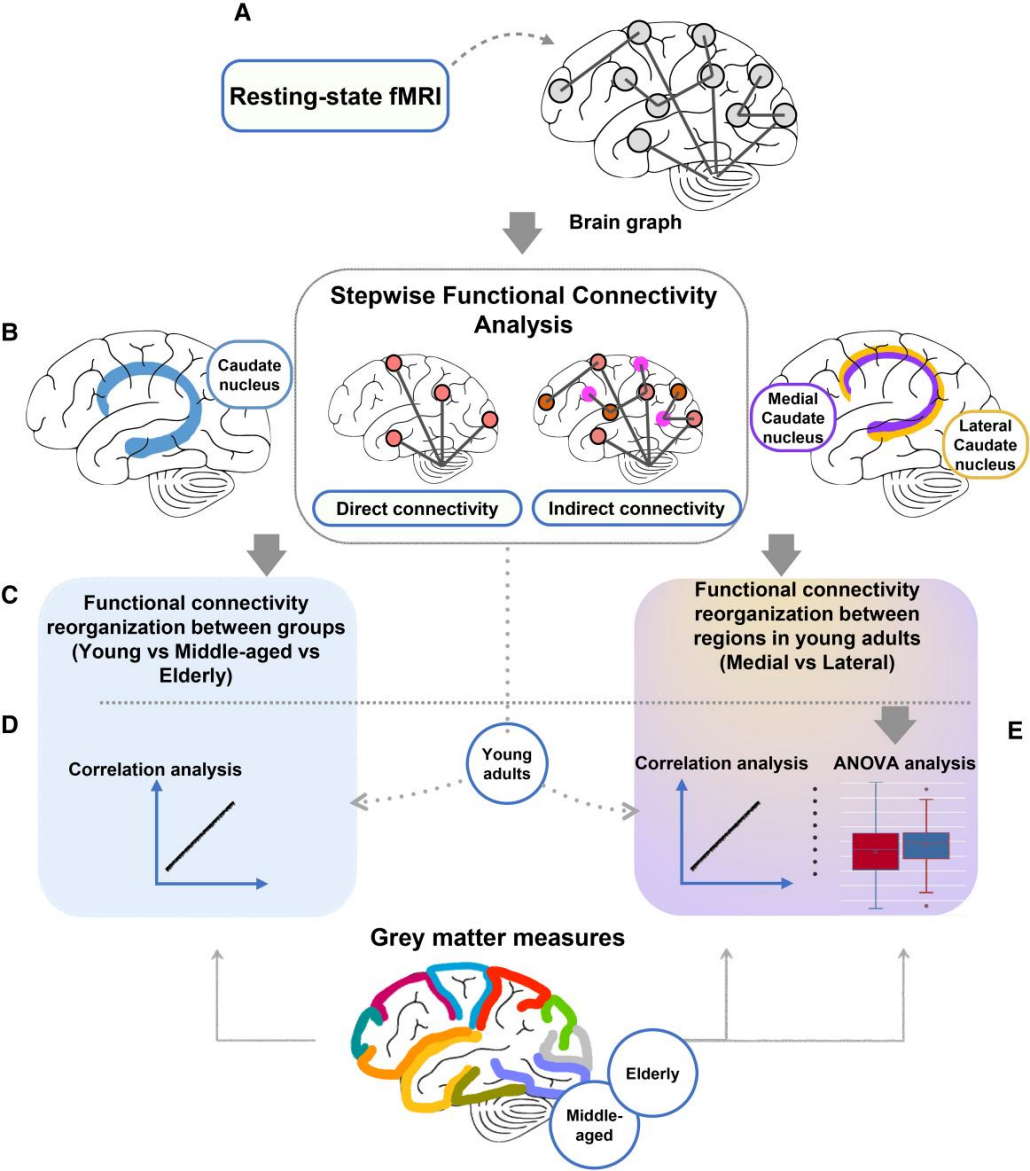
**Study framework.** (**A**) Pipeline of functional connectivity analysis. (**B**) Cortical and subcortical connectivity diagram of the whole caudate and of its subcomponents (medial and lateral) using SFC analysis. Cortical maps show characterization of direct and indirect functional connectivity from different seed regions. (**C**) Functional connectivity reorganization was then evaluated between, firstly, young, middle-aged and elderly subjects and then medial and lateral SFC maps. (**D**) Correlation between direct functional connectivity of each region and the regional standardized GM measures (cortical and subcortical regions) in different groups of healthy subjects. (**E**) ANOVA analysis was performed to evaluate differences of standardized cortical thickness values and GM volumes among regions more connected to the medial caudate than the lateral and regions more connected to the lateral than the medial. fMRI, functional MRI.

Figure 1 reports briefly the study framework: Fig. 1A, pipeline of functional connectivity analysis; Fig. 1B, cortical and subcortical connectivity diagram of the whole caudate and of its subcomponents (medial and lateral) using SFC analysis; Fig. 1C, functional connectivity was then evaluated between (first) young, middle-aged and elderly subjects and (second) medial and lateral SFC maps; Fig. 1D, correlation between direct functional connectivity of each region and the regional standardized GM measures (regional cortical thickness values and GM volumes) in different groups of healthy subjects; and Fig. 1E, ANOVA analysis was performed to evaluate differences of standardized cortical thickness values and GM volumes among regions more connected to the medial or the lateral caudate.

### Socio-demographic and neuropsychological results

Demographics and cognitive characteristics are reported in Table 1 and Supplementary Table 1. Middle-aged subjects differed from both young and elderly participants in terms of age and education, while elderly subjects differ from young subjects in terms of age, sex and education.

### SFC analysis for whole caudate

**Figure 2:**
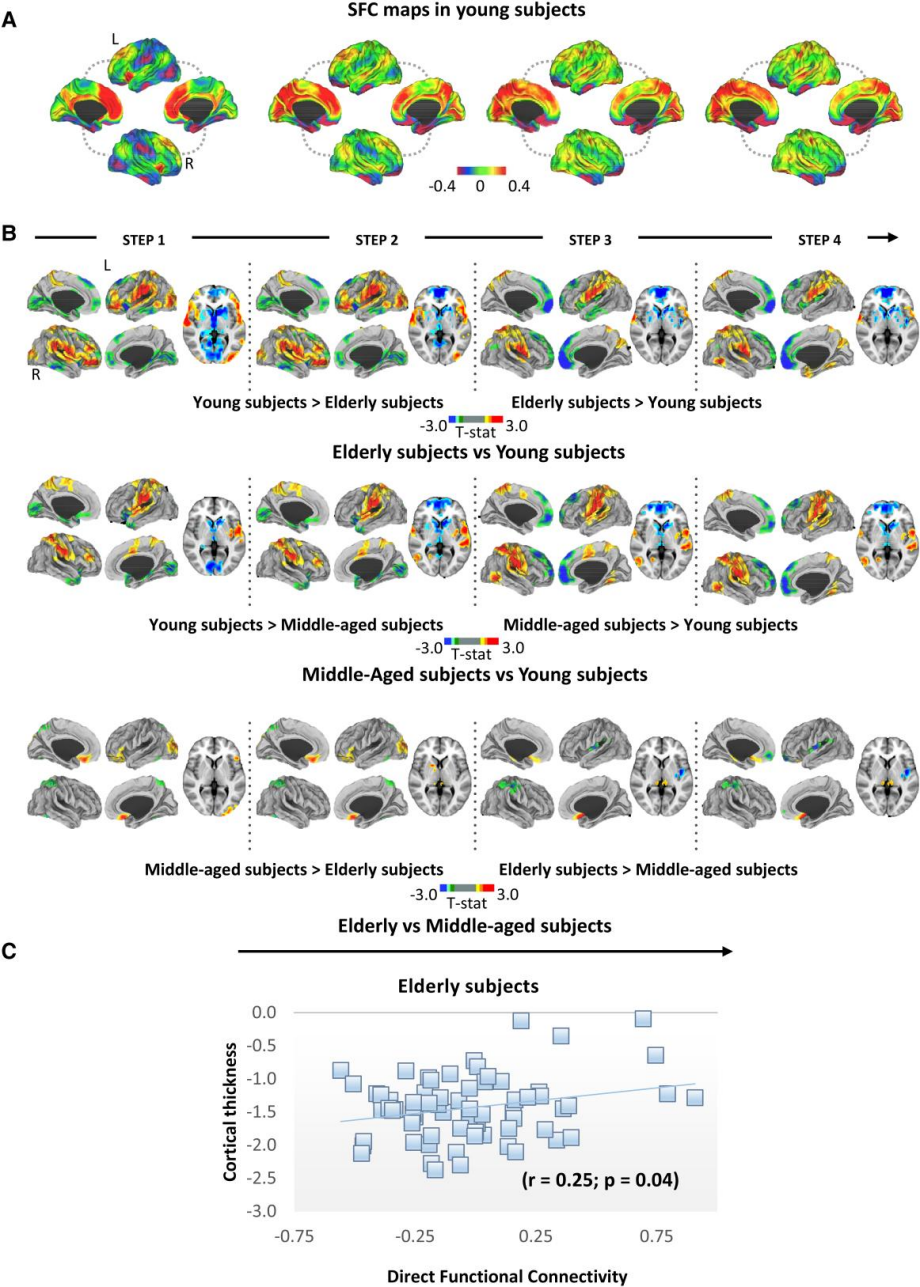
**SFC analysis for whole caudate.** (**A**) Cortical maps represent characterization of stepwise connectivity analysis from the caudate in young healthy subjects. (**B**) Cortical maps represent the significant differences in SFC values between young, middle-aged and elderly subjects. Statistical analysis was adjusted for sex and education. Results were corrected for multiple comparisons using a threshold-free cluster enhancement method combined with nonparametric permutation testing at *P* < 0.05 family-wise error corrected. Colour bars show the *t*-statistic applicable to the image. (**C**) Correlation between SFC of each region at the first-link step in young healthy subjects, as a measure of the strength of direct connectivity, and the regional standardized GM measures (cortical and subcortical regions) in elderly subjects. L, left; R, right; SFC, stepwise functional connectivity.

Investigating the average maps of SFC in the young healthy subjects, we found that the caudate exhibited a dense and predominant regional–local direct functional connectivity (Fig. 2A). Particularly, caudate seed revealed a dense pattern of direct connections reaching firstly medial frontal lobe, cingulate cortex, medial parietal cortex and insula (Fig. 2A). Looking at the indirect connectivity (intermediate steps), caudate seed reached additionally the paracentral lobules, occipital lobe (cuneus and pericalcarine), superior temporal and inferior parietal regions (Fig. 2A). Sensorimotor cortices were reached only in the last steps (Fig. 2A). When comparing the three groups of healthy adults, we found significant differences in the caudate SFC in all four-link step (Fig. 2B; Supplementary Tables 2–4). At one-link step distance from the caudate nuclei, middle-aged and elderly adults compared to young subjects showed a decreased SFC in the same areas but with a greater diffusion of damage in elderly relative to middle-aged adults. Specifically, regions involved were in the frontal lobe (superior frontal and medial orbitofrontal cortex), anterior insula, parietal lobe (precuneus), temporal lobe (fusiform gyri, middle and inferior temporal gyri, entorhinal cortex, parahippocampal cortex and temporal pole), occipital lobe (lingual gyri and pericalcarine), isthmus cingulate cortex, subcortical regions (putamen, caudate and thalamus) and cerebellum. Additional reduced indirect SFC was found in middle-aged and elderly compared to young healthy adults in both superior frontal and medial orbitofrontal cortex. Concerning the opposite contrast, elderly subjects exhibited a significantly increased SFC at one-link step in frontal lobe (lateral orbitofrontal cortex, precentral gyri, rostral middle frontal gyri, pars orbitalis, pars triangularis and pars opercularis), posterior insula, parietal lobe (postcentral gyri, superior and inferior parietal gyri and supramarginal gyri), temporal lobe (superior temporal gyri, posterior middle and inferior temporal gyri—close to the temporal–parietal junction) and occipital lobe (lateral occipital cortex) compared to young subjects. Similarly, middle-aged subjects showed a significantly increased SFC compared to young subjects in the same brain areas of elderly subjects but with a slight preservation of frontal (pars orbitalis, pars triangularis and pars opercularis) and occipital lobes (lateral occipital cortex). Moreover, middle-aged and elderly adults were characterized by increased indirect SFC relative to young adults in fusiform and lingual gyri. At one-link step distance from the caudate nuclei, elderly adults showed a decreased SFC relative to middle-aged subjects in the parietal lobe (superior parietal gyrus and precuneus). Additional reduced indirect SFC was found in elderly adults compared to middle-aged healthy adults in both posterior insula and postcentral gyri and subcortical regions (putamen and pallidum). Concerning the opposite contrast, elderly subjects exhibited a significantly increased SFC at direct and indirect step in frontal lobe (medial and lateral orbitofrontal cortex) and occipital lobe (lateral occipital cortex) relative to middle-aged healthy adults.

### SFC analysis for caudate subcomponents in young subjects

**Figure 3:**
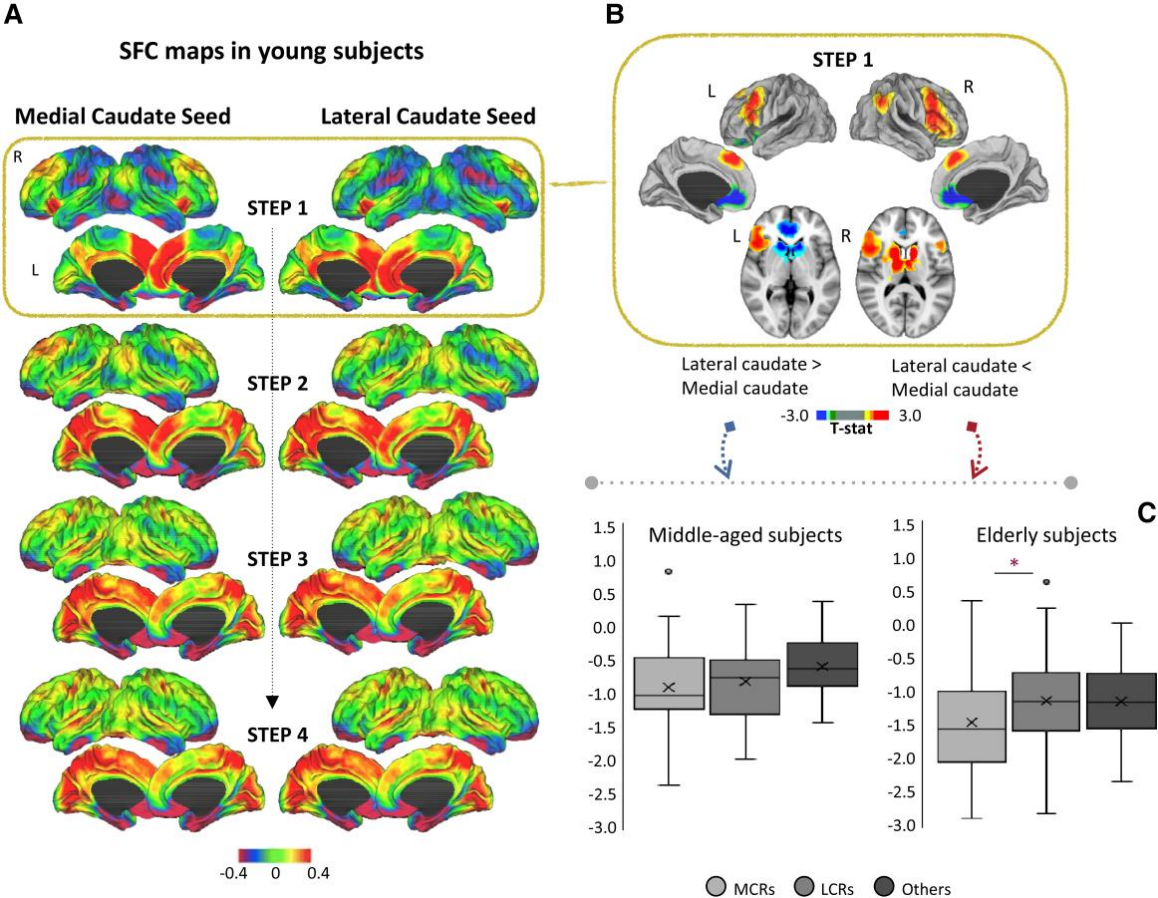
**SFC analysis for caudate subcomponents in young subjects.** (**A**) Cortical maps represent characterization of stepwise connectivity analysis from the medial and lateral caudate seeds in young healthy subjects. (**B**) Cortical maps represent the significant differences in the first-link step of SFC maps between medial and lateral SFC maps. Results were corrected for multiple comparisons using a threshold-free cluster enhancement method combined with nonparametric permutation testing at *P* < 0.05 family-wise error corrected. Colour bars show the *t*-statistic applicable to the image. (**C**) GM analysis for regions connected to the caudate subcomponents. ANOVA analysis was performed to evaluate differences of standardized cortical thickness values and GM volumes among regions more connected to the medial caudate than the lateral and regions more connected to the lateral than the medial (see **B**) in middle-aged and elderly healthy subjects. L, left; LCRs, lateral caudate connected regions; MCRs, medial caudate connected regions; R, right; SFC, stepwise functional connectivity.

Considering the average SFC maps in young subjects, we found that connectivity of medial and lateral caudate seeds was roughly the same and similar to that of the whole caudate. Both medial and lateral caudate nuclei seed regions exhibited dense connectivity with medial frontal lobe, cingulate cortex, medial parietal cortex and insula (Fig. 3A). At the intermediate step, both medial and lateral caudate nuclei seeds revealed an indirect pattern of connectivity to sensorimotor areas, inferior parietal lobule, superior temporal and medial occipital cortical regions (Fig. 3A). When comparing the connectivity of medial and lateral caudate seeds in young subjects, we found significant differences only in the first-link step of SFC maps (Fig. 3B). At one-link step distance, the medial caudate seed shows stronger connectivity with frontal lobe (superior frontal cortex, rostral and caudal middle frontal cortex, pars opercularis, pars triangularis, pars orbitalis and precentral gyri), parietal lobe (inferior parietal gyrus) and anterior insula. In subcortical areas, the medial caudate seed showed stronger connectivity with thalamus, superior caudate and putamen (Fig. 3B). For clarity, all the above-cited regions that had stronger connectivity with the medial caudate were named medial caudate connected regions (MCRs). Concerning the opposite contrast, in young subjects, the lateral caudate nucleus seed exhibited significantly stronger connectivity at one-link step with frontal lobe (orbitofrontal) and rostral anterior cingulate (Fig. 3B). In subcortical areas, lateral caudate seed showed stronger connectivity with inferior caudate and putamen, thalamus and pallidum (Fig. 3B). For clarity, all the above-cited regions that had stronger connectivity with the lateral caudate were named lateral caudate connected regions (LCRs).

### GM structural changes of regions connected to the caudate subcomponents in middle-aged and elderly adults

As expected, GM measures of both MCRs and LCRs (cortical and subcortical) were significantly decreased in middle-aged and elderly adults relative to young adults (*P* < 0.001). Furthermore, lower GM measures of both MCRs (*P* = 0.001) and LCRs (*P* = 0.02) were found in elderly relative to middle-aged adults.

ANOVA analyses in middle-aged subjects comparing GM measures of MCRs, LCRs and all the remaining regions of the brain showed no differences (Fig. 3C). On the contrary, in elderly subjects, MCRs were more atrophic than LCRs, while MCRs and LCRs did not differ from the remaining regions of the brain (Fig. 3C).

### Spatial similarities between caudate SFC in young subjects and GM measures with aging

Considering the spatial similarity between SFC combined map of caudate (whole, medial and lateral) in young subjects and GM measures (cortical and subcortical regions) in middle-aged and elderly, no significant relationship was observed.

Considering direct functional connectivity (one-link step distance) from caudate (whole, medial and lateral), no significant results were found in the correlation with GM measures (cortical and subcortical regions) of middle-aged subjects. However, in elderly adults, direct functional connectivity of whole caudate (*r* = 0.25; *P* = 0.04; Fig. 2C) and lateral caudate (*r* = 0.31; *P* = 0.01) presented a significant positive correlation with regional cortical thickness values. In elderly subjects’ group, no correlation was found between direct functional connectivity and GM volumes.

## Discussion

The present study aimed to investigate how the functional organization of the caudate network relates to structural brain changes with aging. We applied the SFC technique to RS-fMRI of healthy young, middle-aged and elderly subjects. Our findings revealed a reorganization of the caudate networks through the aging process. In elderly subjects, functional connectivity of the direct connections to the caudate nuclei showed a positive correlation with cortical thickness measures. Importantly, in these subjects, cortical regions connected to the medial caudate were thinner than those linked to the lateral caudate, suggesting that brain structural changes might be differently shaped by the medio-lateral gradient of connections with the caudate nucleus due to the age-related periventricular changes, including vascular, degenerative and inflammatory alterations and a possible SVZ neurogenesis decay.

### Caudate functional connectivity with aging

The striatum presents extensive connections with almost the entire cerebral cortex.^30^ Previous studies have already described the topographical organization of caudate connections.^10,31^ Moreover, the topological embedding of the striatum and thalamus within the global brain connectivity network suggests that they belong to an exclusive rich-club circuit and are key regions for communication across large-scale network systems^32^ and integration of information.^33^ Despite its importance, just a few studies analysed the effect of aging on caudate connectivity.^2,12^ In this study, we confirmed that caudate connections actually reach almost the entire cortex in a few functional steps. With aging, brain regions that presented altered functional connectivity with caudate were consistent over the four-link step distances (direct and indirect connectivity).

Relative to young healthy adults, middle-aged and elderly adults showed that caudate reduced its functional connectivity with key regions included in the frontoparietal control network and in the DMN, such as superior frontal gyri, medial orbitofrontal cortex, medial parietal (precuneus) and lateral temporal (middle and inferior) regions. The results of our study are mostly in line with previous findings,^2^ with slight inconsistencies being probably explained by differences in the caudate subdivisions. Importantly, however, our results have emphasized a possible loss of selective organization of the functional cortical–caudate network, particularly frontoparietal and DMN, with aging. Indeed, it has been demonstrated that those functional cortical networks are commonly altered with aging and related to cognitive decline, in particular with memory deficits.^2^ Interestingly, our findings revealed spatial similarities when comparing young and middle-aged adults with elderly adults. However, a more extensive pattern of damage was observed in the elderly relative to the young subjects. This emphasizes that aging is a gradual process, unfolding over time, primarily affecting distributed fronto–temporo–parietal areas.^5^

On the contrary, caudate nucleus increased its connectivity with sensorimotor areas and posterior middle temporal gyrus (outside DMN) already in middle-aged relative to young adults, but not with visual cortex (altered only in elderly subjects). Increased connectivity can be interpreted as a compensatory strategy for the decreased link between caudate and brain regions belonging to frontoparietal control network and DMN in middle-aged and elderly adults.^2^ The delayed alteration in connectivity with the visual cortex, specifically emerging in elderly subjects, might be associated with age-related changes in visual processing. Visual functions tend to exhibit more pronounced decline in later life stages, possibly reflecting a cumulative impact of aging on the visual system. Therefore, the observed connectivity changes in the elderly might signify a manifestation of age-related adjustments in the neural networks associated with visual perception.^34^ An increased functional connectivity of the caudate with middle temporal gyrus can reflect a lower decoupling between those two regions with aging, which has been associated with memory decline.^2^

### The caudate network and cortical thinning with aging

We supposed that caudate nucleus activity and its functional network is important in shaping aging-associated structural alterations of the human brain. Our results showed that direct functional connectivity with the caudate was positively related to cortical thickness measures in elderly subjects. It would seem that having a strong direct functional connectivity at one-link step distance with the caudate is beneficial for the cortex. The caudate nucleus is able to send feedback signals to the cortex; thus, it influences and regulates cortical activity. This signal could be helpful in modulating/dampening/tightly regulating excitatory signals if needed.^12^ A chronic imbalance of excitatory synapses in the cortex might lead to excitotoxicity and with time to macroscopical atrophy. It is plausible that such imbalance is present in the cortex of elderly subjects given also the numerous aging-associated microscopical changes—lacunes, microinfarcts and protein deposits.

### Medial and lateral caudate functional networks and the relationship with age-related structural changes

Subregions of the caudate nucleus might suffer from different influences over time in normal aging and thus alter its functionality to the cortex. The part of the caudate in contact with the ventricle is characterized by a different vascular supply than the lateral one,^35^ thus being exposed to a possible different vascular damage over time. Moreover, the adjacency to the lateral ventricle might expose this region to toxic metabolites present in the periventricular regions due to transependymal flow.^13,36^ The medial caudate might also resent of changes of the SVZ due to the evolution of the neural stem, astrocytic and vascular compartment.^14^ Indeed, our results suggested that functional direct connectivity of the medial caudate (directly in contact with the ventricle) to other brain regions is different from that of the lateral caudate. Particularly, medial caudate was linked to dorsolateral frontal and parietal and superior medial frontal regions, while lateral caudate was more connected to ventral brain regions. Such result was in line with previous findings suggesting a relation between medial–lateral gradient of the caudate and functional cortical networks.^12,37^ This caudate organization might help to integrate internal information, linked to ventral regions and DMN and external information processed by attention networks.^12,37^

Concerning the influences of the two caudate subregions on structural brain integrity, we found that in elderly subjects, MCRs showed reduced thickness compared to LCRs. Our hypothesis is that MCRs suffer from the selective influence of the SVZ on the medial caudate. An interesting recent discovery might even suggest a direct effect of the SVZ in regulating striatal activity through the secretion of soluble molecules that hypothetically have a diffusion gradient along the medial–lateral direction.^14^ However, microstructural damage accumulates with aging in these neurogenic areas of the brain^38^ leading to altered SVZ functionality and medial caudate activity.^20^ Ultimately, the altered medial caudate activity might result in deregulation of excitatory–inhibitory balance in the cortex. Given the fact that most MCRs belong to the frontal lobe, our results support the fronto-striatal network degeneration in aging.^39-41^ Functional connectivity of the medial caudate influenced the deterioration of MCRs with aging, but not of the whole brain.

### Limitations

The study is not without limitations. First, the split of the 152 normal subjects into discrete categories (young, middle-aged and elderly) using 35 and 60 years of age as cut-off might be arbitrary, and future studies are needed to confirm our findings. Second, although the neuropsychological characterization of our participants was very comprehensive, there is a lack of information about lifestyle risk factors (i.e. smoking, obesity, lifestyle and health risk factors). Another limitation lies in the cross-sectional nature of the study. In this context, longitudinal studies are warranted to verify the trajectories of functional changes, assessing the evolution of alterations over time. Regarding methodological concerns, computational constraints required us to down sample data to relatively large voxels (5 mm^3^). Moreover, SFC analysis does not provide information on the directionality of the functional connectivity. Segmentation of subcortical structures is usually less precise than cortical one, and the variability in ventricle dimension could impair caudate assessment. Finally, one major difference between our gradient mapping approach from others is that we used the criterion of the adjacency to the ventricles for the subdivision of the caudate nucleus. For this reason, the comparison with previous studies can be challenging.

## Conclusion

In conclusion, our study pointed out the role of the caudate nucleus functional network in the aging process and suggested that brain periventricular changes, including SVZ alterations, may have a role in age-related network disruption in humans. The evaluation of the caudate nucleus with RS-fMRI might help to better characterize the aging phenotype and possibly lead to prevention strategies to preserve cognitive functions.

## Supplementary Material

fcae116_Supplementary_Data

## Data Availability

The data set used and analysed during the current study will be made available by the corresponding author upon request to qualified researchers (i.e. affiliated to a university or research institution/hospital).
