# Supplementary material for: Caudate functional networks influence brain structural changes with aging
Source: Brain Commun. 2024 Apr 9;6(2):fcae116. doi: 10.1093/braincomms/fcae116 (PMC11043654; doi:10.1093/braincomms/fcae116)
Supplement: fcae116_Supplementary_Data [file fcae116_supplementary_data.docx]

**SUPPLEMENTARY METHODS**

**Neuropsychological assessment**

In all subjects, neuropsychological assessment evaluated: global cognition with the Mini-Mental State Examination (MMSE);^1^ memory with the Rey Auditory Verbal Learning test^2^ and the delayed recall of a complex figure(Rey–Osterrieth^3^ or Benson^4^); attention and executive functions with the Trail Making Test^5^ and the Modified Card Sorting Test;^6^ visuospatial abilities with the copy of a complex figure (Rey–Osterrieth^3^ or Benson^4^); and mood with the Beck Depression Inventory.^7^ In addition, in older adults we further assessed memory with the digit and spatial span forward;^8^ attention and executive functions with the attentive matrices,^9^ the Raven coloured progressive matrices,^10^ the digit span backward,^11^ and the phonemic and semantic fluencies;^12^ visuospatial abilities with the freehand copying drawings with or without guiding landmarks;^2^ language with the Token Test;^13^ and behaviour with the apathy rating scale.^14^ On the other hand, in young adults we also assessed attention and executive functions with the Pasat 2” test;^15^ visuospatial abilities with the Benton line orientation test;^16^ language with naming in response to an oral description (CaGi);^17^ and physical and mental wellness with the Symptom Check List-90-Revised (SCL-90-R).^18^

**Resting state (RS) fMRI analysis**

**Pre-processing.** Analysis of the RS-fMRI data was performed with Data Processing Assistant for Resting-State toolbox (DPARSFA, http://rfmri.org/ DPARSF,^19^ based on Statistical Parametric Mapping (SPM12, http://www.fil.ion.ucl.ac.uk/spm), and the RS-fMRI Data Analysis Toolkit [http://www.restfmri.net].^20^ Pre-processing included the following steps: removal of the first four volumes of raw RS-fMRI data, slice timing correction (the middle slice was used as the reference point), head motion correction applying a six-parameter (rigid body) linear transformation and a two-step procedure by registering to the first image and then to the mean of the images after the first realignment), spatial normalization to the Montreal Neurological Institute (MNI) atlas template with voxel size set at 5 × 5 × 5 mm^3^ for computational efficiency, removal of spurious variance through linear regression. This step included 24 parameters from the head motion correction step [6 head motion parameters, 6 head motion parameters one time point before, and the 12 corresponding squared items],^21^ scrubbing with regression [signal spike regression as well as 1 back and 2 forward neighbors]^22^ at time points with a frame-wise displacement (FD)>0.5mm,^23^ linear and quadratic trends, global signal, white matter signal, and the cerebrospinal fluid signal. The last steps of preprocessing included spatial smoothing with a 4 mm FWHM Gaussian Kernel and band-pass temporal filtering (0.01-0.08 Hz) to reduce the effect of low frequency drift and high frequency noise.^24^

No participant had more than 2 mm/degree of movement in any of the six directions, and no more than 8 volumes removed during scrubbing (1/3 of the total volumes), ensuring at least 5 minutes and 30 seconds of functional data per individual.

**Functional connectome reconstruction.** As input to the SFC analysis, we first computed the individual association matrices for each participant by assessing the Pearson correlation of each voxel to every other voxel time course within a mask covering cortical gray matter. Pre-processed RS-fMRI data of each participant were previously converted to an N-by-M matrix, where N was the image voxels in MNI space, and M was the 320 acquisition time points. From this step, a 11705x11705 matrix of Pearson correlation coefficients (i.e., r-values) was obtained per each participant. Fisher z transformation was applied to r-values. Then, all negative correlations and positive correlations that did not reach any false discovery rate (FDR) correction threshold of p<0.05 were excluded from further analyses.^25^ Only positive correlations of the association matrix were taken into account, as positive connectivity has been proved to drive functional connectivity network topology in the human brain.^26^

**Stepwise functional connectivity analysis.** SFC analysis is a graph-theory-based method that aims to characterize regions that connect to specific seed brain areas at different levels of “link-step” distances. Therefore, it allows to detect both direct and indirect functional couplings of a given seed region to other regions in the brain.^26-28^ With such a framework, a step refers to the number of links (edges) that belongs to a path connecting a node to the seed (or target) area. Accordingly, link step and path length are analogous concepts. In SFC analysis, the degree of stepwise connectivity of a voxel *j* for a given step distance *l* and a seed area *i* () is computed from the count of all paths that (1) connect voxel *j* and any voxel in seed area *i*, and (2) have an exact length of *l*. Each SFC matrix of size m-by-m can be recursively represented as follows:

*Equation 1:*

Here, is the functional connectivity matrix with a step distance of *l*, and is the correlation matrix after Fisher transformation. Matrices were then normalized between 0 and 1, keeping the final distribution of values intact while making them comparable across step distances. In this sense, a larger SFC degree under the step distance *l* indicates stronger paths connecting two voxels via link one, while a smaller degree indicates weaker connectivity paths. We explored a wide range of link-step distances, from 1 to 20, to characterize the progression of the derived maps. Although the amount of overlap between consecutive steps is expected to be high, we aimed to see meaningful relative changes between pairs of maps. SFC patterns are topographically dissimilar between consecutive maps from steps one to three and become stable for link-step distances above four. Based on this analysis, in our results we show only maps up to four steps. Furthermore, we refer to functional connectivity at one-link step as direct connectivity and for the functional connectivity at subsequent steps (2-4) as indirect connectivity.

**Supplementary Table 1.** Comprehensive neuropsychological findings in young, middle-aged and elderly subjects.

|  |  | **Young healthy subjects** | **Middle-aged subjects** | **Elderly**  **subjects** | **p-value Young**  ***vs***  **Middle-aged subjects** | **p-value Young**  ***vs***  **Elderly subjects** | **p-value Middle-aged**  ***vs***  **Elderly subjects** |
| --- | --- | --- | --- | --- | --- | --- | --- |
| **Global cognition** | MMSE | 29.84 ± 0.37  (29.00 – 30.00) | 29.5 ± 0.66  (28.00 – 30.00) | 29.23 ± 0.93  (27.00 – 30.00) | 0.16 | **<0.001** | 0.40 |
| **Memory** | Digit Span, forward | - | 5.96 ± 1.12  (4.00 – 8.00) | 5.92 ± 0.88  (4.00 – 8.00) | - | - | 0.89 |
|  | Rey’s figure [recall] | 23.51 ± 4.77  (11.00 – 33.00) | 18.87 ± 5.33  (7.00 – 23.00) | 13.35 ± 5.50  (6.50 – 23.00) | 0.60 | **<0.001** | 0.27 |
|  | Benson’s figure [recall] | - | 11.62 ± 2.82  (8.00 – 17.00) | 10.00 ± 3.37  (4.00 – 14.00) | 0.15 | - | 0.15 |
| **Attention and executive functions** | Trail Making Test (B-A) | 37.21 ± 15.42  (14.01 – 105.40) | 54.35 ± 4.21  (19.99 – 88.84) | 72.66 ± 48.97  (33.22 – 209.69) | 0.11 | **<0.001** | 0.10 |
|  | Trail Making Test A | 22.42 ± 6.73  (12.22 − 42.15) | 31.65 ± 7.85  (17.50 − 51.11) | 37.50 ± 14.97  (16.28 − 84.00) | **<0.001** | **<0.001** | 0.12 |
|  | Trail Making Test B | 59.58 ± 17.79  (40.47 − 133.00) | 86.00 ± 22.41  (46.73 – 124.00) | 110.16 ± 60.36  (49.50 – 281.00) | **0.03** | **<0.001** | 0.06 |
|  | Attentive matrices | - | 53.83 ± 5.17  (41.00 − 60.00) | 51.03 ± 7.09  (27.00 – 60.00) | - | - | 0.11 |
|  | Phonemic fluency | - | 33.92 ± 6.47  (21.00 − 46.00) | 39.42 ± 8.68  (25.00 – 59.00) | **-** | - | **0.01** |
|  | Semantic fluency | - | 48.92 ± 9.49  (34.00 – 70.00) | 44.34 ± 9.26  (27.00 – 62.00) | - | - | 0.07 |
|  | Digit span, backward | - | 5.13 ± 1.36  (3.00 − 8.00) | 4.46 ± 0.99  (3.00 – 8.00) | **-** | - | **0.03** |
|  | MCST, categories | 5.49 ± 0.87  (3.00 – 6.00) | 4.50 ± 1.18  (2.00 – 6.00) | 4.34 ± 1.48  (1.00 – 6.00) | **<0.001** | **<0.001** | 1.00 |
|  | MCST, perseverations | 1.27 ± 2.30  (0.00 – 10.00) | 3.42 ± 2.92  (0.00 – 11.00) | 3.84 ± 4.02  (0.00 – 16.00) | **0.02** | **0.001** | 1.00 |
|  | Pasat 2” | 40.92 ± 9.93  (19.00 – 58.00) | - | 29.50 ± 6.36  (25.00 – 34.00) | - | 0.13 | - |
| **Language** | Token Test | - | 34.63 ± 1.13  (32.00 – 36.00) | 33.88 ± 2.03  (28.00 – 36.00) | - | - | 0.11 |
| **Visuospatial** | Rey’s figure [copy] | 32.84 ± 2.12  (26.00 − 36.00) | 30.25 ± 2.38  (26.00 – 32.00) | 28.24 ± 4.03  (19.50 – 33.00) | 0.06 | **<0.001** | 0.27 |
|  | Benson’s figure [copy] | - | 16.00 ± 0.63  (15.00 – 17.00) | 15.59 ± 0.80  (14.00 – 17.00) | - | - | 0.11 |
|  | Copy of drawings [freehand] | - | 10.88 ± 1.03  (9.00 – 12.00) | 9.92 ± 1.91  (4.00 – 12.00) | **-** | **-** | **0.03** |
|  | Copy of drawings with landmarks | - | 67.75 ± 2.72  (60.00 – 70.00) | 66.68 ± 4.30  (51.00 – 70.00) | - | - | 0.28 |
| **Mood**  **and**  **behavior** | Apathy Rating Scale | 5.65 ± 3.80  (0.00 – 16.00) | 6.27 ± 5.29  (0.00 – 22.00) | 6.22 ± 3.88  (0.00 – 15.00) | 1.00 | 1.00 | 1.00 |
|  | SCL-90-R [Total] | 39.80 ± 40.72  (2.00 – 163.00) | - | 10.50 ± 6.36  (6.00 – 15.00) | - | 0.33 | - |

Values are reported as mean ± standard deviation (range). Differences in neuropsychological profile between young, middle-aged and elderly subjects were assessed using one-way ANOVA corrected for age, sex and education (p<0.05). P values were adjusted for Bonferroni- multiple comparison. *Abbreviations*: MCST= Modified Card Sorting test; MMSE= Mini Mental State Examination; SCL-90-R=Symptom Checklist-90-Revised.

**Supplementary Table 2.** Differences between the combined map of the SFC of caudate nuclei between elderly adults and young subjects.

|  | ***STEP 1*** | ***STEP 2*** | ***STEP 3*** | ***STEP 4*** |
| --- | --- | --- | --- | --- |
| ***Elderly subjects vs Young subjects*** | | | | |
| ↑ (yellow-red) | **L&R** pars opercularis, pars orbitalis and pars triangularis, rostral middle frontal gyri and lateral orbitofrontal cortex, precentral, postcentral, supramarginal, superior parietal and superior temporal gyri, lateral occipital cortex;  **L** posterior middle and inferior temporal; **R** posterior insula | **L&R** pars opercularis, pars orbitalis and pars triangularis caudal anterior and posterior cingulate cortex, precentral, postcentral, superior parietal, precuneus, supramarginal and superior temporal gyri; | **L&R** precentral, postcentral, superior and inferior parietal, cuneus, precuneus, supramarginal and superior parietal gyri | **L&R** precentral, postcentral, superior and inferior parietal, precuneus, and supramarginal; **R** cuneus, lingual, fusiform gyri, and pericalcarine |
| ↓ (green-blue) | **L&R** superior frontal gyri, medial orbitofrontal, isthmus cingulate cortex, fusiform, middle and inferior temporal gyri, parahippocampal, lingual gyri, pericalcarine cortex and anterior insula; **L** entorhinal gyri | **L&R** superior frontal and rostral middle frontal gyri and medial orbitofrontal cortex, middle and inferior temporal gyri, and insula; **L** rostral anterior cingulate cortex, parahippocampal and entorhinal gyri; **R** caudal middle frontal | **L&R** superior frontal and rostral middle frontal gyri and medial orbitofrontal cortex, rostral anterior cingulate cortex and insula; **L** middle and inferior temporal gyri | **L&R** superior frontal and rostral middle frontal gyri and medial orbitofrontal cortex and insula; **L** parahippocampal gyrus; **R** rostral and caudal anterior cingulate cortex |
| Subcortical differences | ↓ **L&R** putamen, caudate and hippocampus; **L** amygdala; **R** thalamus; cerebellum | ↓ **L&R** caudate; **L** putamen and pallidum, hippocampus and amygdala; **R** thalamus; cerebellum | ↓ **L&R** caudate; **L** putamen and pallidum, hippocampus and amygdala; **R** thalamus; cerebellum | ↓ **L&R** caudate; **L** putamen and pallidum, hippocampus and amygdala; **R** thalamus; cerebellum |

**Supplementary Table 3.** Differences between the combined map of the SFC of caudate nuclei between middle-aged adults and young subjects.

|  | ***STEP 1*** | ***STEP 2*** | ***STEP 3*** | ***STEP 4*** |
| --- | --- | --- | --- | --- |
| ***Middle-aged subjects vs Young subjects*** | | | | |
| ↑ (yellow-red) | **L&R** precentral, postcentral, paracentral, supramarginal, superior parietal and superior temporal gyri, precuneus;  **L** inferior parietal gyri and posterior insula; **R** rostral middle frontal gyri and posterior cingulate cortex | **L&R** precentral, postcentral, paracentral, supramarginal, superior parietal and superior temporal gyri, precuneus;  **L** inferior parietal gyri and posterior insula; **R** rostral middle frontal gyri and posterior cingulate cortex | **L&R** precentral, postcentral, paracentral, supramarginal, superior parietal and superior temporal gyri, precuneus and posterior insula; **R** fusiform gyrus, lateral occipital and posterior cingulate cortex | **L&R** precentral, postcentral, supramarginal, superior parietal and superior temporal gyri, precuneus gyri and posterior insula;  **R** fusiform and lingual gyri, lateral occipital and posterior cingulate cortex |
| ↓ (green-blue) | **L&R** medial orbitofrontal, fusiform, middle and inferior temporal gyri, temporal pole, parahippocampal, lingual gyri, pericalcarine cortex and anterior insula; entorhinal gyri | **L&R** medial orbitofrontal cortex, superior, middle and inferior temporal gyri, and anterior insula, parahippocampal and entorhinal gyri, lingual and pericalcarine gyri, cuneus | **L&R** superior frontal and rostral middle frontal gyri and medial orbitofrontal cortex, and anterior insula | **L&R** superior frontal and rostral middle frontal gyri and medial orbitofrontal cortex, and anterior insula |
| Subcortical differences | ↓ **L&R** caudate; **R** thalamus; cerebellum;  ↑ **R** putamen | ↓ **L&R** caudate; **L** putamen and pallidum; **R** thalamus; cerebellum;  ↑ **R** putamen | ↓ **L&R** caudate; **R** thalamus; cerebellum;  ↑ **R** putamen | ↓ **L&R** caudate; **R** thalamus; cerebellum;  ↑ **R** putamen |

**Supplementary Table 4.** Differences between the combined map of the SFC of caudate nuclei between elderly adults and middle-aged subjects.

|  | ***STEP 1*** | ***STEP 2*** | ***STEP 3*** | ***STEP 4*** |
| --- | --- | --- | --- | --- |
| ***Elderly subjects vs Middle-aged subjects*** | | | | |
| ↑ (yellow-red) | **L&R** medial orbitofrontal cortex; **L** lateral orbitofrontal cortex, pars orbitalis and lateral occipital cortex | **L&R** medial orbitofrontal cortex; **L** lateral orbitofrontal cortex, pars orbitalis and lateral occipital cortex | **L&R** medial orbitofrontal cortex;  **L** parahippocampal gyrus | **L&R** medial orbitofrontal cortex;  **L** parahippocampal gyrus |
| ↓ (green-blue) | **L&R** precuneus and superior parietal gyri | **L&R** precuneus and superior parietal gyri | **L** insula;  **R** superior parietal and postcentral gyri | **L** superior temporal gyrus and insula;  **R** superior parietal and postcentral gyri |
| Subcortical differences | - | ↑ **L** pallidum and putamen | ↓ **R** putamen | ↓ **R** putamen |

**Supplementary Table 5.** MRI acquisition parameters.

| **Milan** | Philips Medical System Ingenia CX 3T scan | | | |
| --- | --- | --- | --- | --- |
|  | 3D T2-weighted | 3D fluid-attenuated inversion recovery | 3D high resolution T1-weighted | T2*-weighted single-shot EPI sequence  (RS-fMRI) |
| **Repetition time (msec)** | 2500 | 4800 | 7 | 1567 |
| **Echo time (msec)** | 330 | 267 | 3.2 | 35 |
| **Flip angle** | - | 90° | 9° | 70° |
| **Section thickness (mm)** | 1 | 1 | 1 | 3 |
| **No. of sections** | 192 | 192 | 204 | 48 for 320 volumes |
| **Matrix** | 256x256 | 256x256 | 256x240 | - |
| **Field of view (mm^2^)** | 256x256 | 256x256 | 256x240 | 240x240 |

*Abbreviations*: FFE= fast field echo; FLAIR= fluid-attenuated inversion recovery; MRI= magnetic resonance imaging; msec= millisecond; mm= millimeter; No= number; RS fMRI= resting state functional MRI; SE=spin echo; sec=second.

**REFERENCES**

1. Folstein MF, Folstein SE, McHugh PR. "Mini-mental state". A practical method for grading the cognitive state of patients for the clinician. *J Psychiatr Res*. Nov 1975;12(3):189-98. doi:10.1016/0022-3956(75)90026-6

2. Carlesimo GA, Caltagirone C, Gainotti G. The Mental Deterioration Battery: normative data, diagnostic reliability and qualitative analyses of cognitive impairment. The Group for the Standardization of the Mental Deterioration Battery. *Eur Neurol*. 1996;36(6):378-84. doi:10.1159/000117297

3. Caffarra P, Vezzadini G, Dieci F, Zonato F, Venneri A. Rey-Osterrieth complex figure: normative values in an Italian population sample. *Neurol Sci*. Mar 2002;22(6):443-7. doi:10.1007/s100720200003

4. Possin KL, Laluz VR, Alcantar OZ, Miller BL, Kramer JH. Distinct neuroanatomical substrates and cognitive mechanisms of figure copy performance in Alzheimer's disease and behavioral variant frontotemporal dementia. *Neuropsychologia*. Jan 2011;49(1):43-8. doi:10.1016/j.neuropsychologia.2010.10.026

5. Giovagnoli AR, Del Pesce M, Mascheroni S, Simoncelli M, Laiacona M, Capitani E. Trail making test: normative values from 287 normal adult controls. *Italian journal of neurological sciences*. Aug 1996;17(4):305-9. doi:10.1007/BF01997792

6. Caffarra P, Vezzadini G, Dieci F, Zonato F, Venneri A. Modified Card Sorting Test: normative data. *J Clin Exp Neuropsychol*. Apr 2004;26(2):246-50. doi:10.1076/jcen.26.2.246.28087

7. Beck AT, Ward CH, Mendelson M, Mock J, Erbaugh J. An inventory for measuring depression. *Arch Gen Psychiatry*. Jun 1961;4:561-71. doi:10.1001/archpsyc.1961.01710120031004

8. Orsini A, Grossi D, Capitani E, Laiacona M, Papagno C, Vallar G. Verbal and spatial immediate memory span: normative data from 1355 adults and 1112 children. *Italian journal of neurological sciences*. Dec 1987;8(6):539-48. doi:10.1007/BF02333660

9. [Italian standardization and classification of Neuropsychological tests. The Italian Group on the Neuropsychological Study of Aging]. *Italian journal of neurological sciences*. Dec 1987;Suppl 8:1-120. Standardizzazione e taratura italiana di test neuropsicologici. Gruppo Italiano per lo Studio Neuropsicologico dell'Invecchiamento.

10. Basso A, Capitani E, Laiacona M. Raven's coloured progressive matrices: normative values on 305 adult normal controls. *Functional neurology*. Apr-Jun 1987;2(2):189-94.

11. Monaco M, Costa A, Caltagirone C, Carlesimo GA. Forward and backward span for verbal and visuo-spatial data: standardization and normative data from an Italian adult population. *Neurol Sci*. May 2013;34(5):749-54. doi:10.1007/s10072-012-1130-x

12. Novelli G, Papagno C, Capitani E, Laiacona M, et al. Tre test clinici di memoria verbale a lungo termine: Taratura su soggetti normali. [Three clinical tests for the assessment of verbal long-term memory function: Norms from 320 normal subjects.]. *Archivio di Psicologia, Neurologia e Psichiatria*. 1986;47(2):278-296.

13. De Renzi E, Vignolo LA. The token test: A sensitive test to detect receptive disturbances in aphasics. *Brain*. Dec 1962;85:665-78. doi:10.1093/brain/85.4.665

14. Marin RS, Biedrzycki RC, Firinciogullari S. Reliability and validity of the Apathy Evaluation Scale. *Psychiatry Res*. Aug 1991;38(2):143-62. doi:10.1016/0165-1781(91)90040-v

15. Amato MP, Portaccio E, Goretti B, et al. The Rao's Brief Repeatable Battery and Stroop Test: normative values with age, education and gender corrections in an Italian population. *Mult Scler*. Dec 2006;12(6):787-93. doi:10.1177/1352458506070933

16. Benton AL, Varney NR, Hamsher KD. Visuospatial judgment. A clinical test. *Arch Neurol*. Jun 1978;35(6):364-7. doi:10.1001/archneur.1978.00500300038006

17. Catricala E, Della Rosa PA, Ginex V, Mussetti Z, Plebani V, Cappa SF. An Italian battery for the assessment of semantic memory disorders. *Neurol Sci*. Jun 2013;34(6):985-93. doi:10.1007/s10072-012-1181-z

18. Derogatis LR, Savitz KL. The SCL–90–R and Brief Symptom Inventory (BSI) in primary care. *Handbook of psychological assessment in primary care settings*. Lawrence Erlbaum Associates Publishers; 2000:297-334.

19. Chao-Gan Y, Yu-Feng Z. DPARSF: A MATLAB Toolbox for "Pipeline" Data Analysis of Resting-State fMRI. *Front Syst Neurosci*. 2010;4:13. doi:10.3389/fnsys.2010.00013

20. Song XW, Dong ZY, Long XY, et al. REST: a toolkit for resting-state functional magnetic resonance imaging data processing. *PLoS One*. 2011;6(9):e25031. doi:10.1371/journal.pone.0025031

21. Friston KJ, Williams S, Howard R, Frackowiak RS, Turner R. Movement-related effects in fMRI time-series. *Magn Reson Med*. Mar 1996;35(3):346-55. doi:10.1002/mrm.1910350312

22. Yan CG, Craddock RC, He Y, Milham MP. Addressing head motion dependencies for small-world topologies in functional connectomics. *Front Hum Neurosci*. 2013;7:910. doi:10.3389/fnhum.2013.00910

23. Jenkinson M, Bannister P, Brady M, Smith S. Improved optimization for the robust and accurate linear registration and motion correction of brain images. *Neuroimage*. Oct 2002;17(2):825-41. doi:10.1016/s1053-8119(02)91132-8

24. Biswal B, Yetkin FZ, Haughton VM, Hyde JS. Functional connectivity in the motor cortex of resting human brain using echo-planar MRI. *Magn Reson Med*. Oct 1995;34(4):537-41. doi:10.1002/mrm.1910340409

25. Benjamini Y, Drai D, Elmer G, Kafkafi N, Golani I. Controlling the false discovery rate in behavior genetics research. *Behav Brain Res*. Nov 1 2001;125(1-2):279-84. doi:10.1016/s0166-4328(01)00297-2

26. Qian J, Diez I, Ortiz-Teran L, et al. Positive Connectivity Predicts the Dynamic Intrinsic Topology of the Human Brain Network. *Front Syst Neurosci*. 2018;12:38. doi:10.3389/fnsys.2018.00038

27. Gao Q, Yu Y, Su X, et al. Adaptation of brain functional stream architecture in athletes with fast demands of sensorimotor integration. *Hum Brain Mapp*. Feb 1 2019;40(2):420-431. doi:10.1002/hbm.24382

28. Sepulcre J, Sabuncu MR, Yeo TB, Liu H, Johnson KA. Stepwise connectivity of the modal cortex reveals the multimodal organization of the human brain. *J Neurosci*. Aug 1 2012;32(31):10649-61. doi:10.1523/JNEUROSCI.0759-12.2012
